# Mothers’ perspectives on the perinatal loss of a co-twin: a qualitative study

**DOI:** 10.1186/s12884-015-0579-z

**Published:** 2015-07-02

**Authors:** Judy Richards, Ruth Graham, Nicholas D Embleton, Claire Campbell, Judith Rankin

**Affiliations:** Institute of Health & Society, Newcastle University, Baddiley-Clark Building, Richardson Road, Newcastle upon Tyne, England, NE2 4AE UK; School of Geography, Politics and Sociology, Newcastle University, Newcastle upon Tyne, England, NE1 7RU UK; Newcastle Hospitals NHS Foundation Trust, Newcastle Neonatal Service, Newcastle upon Tyne, England, NE2 4AE UK

**Keywords:** Bereavement, Multiple pregnancy, Twins, Grief, Trauma

## Abstract

**Background:**

There is a growing body of literature exploring the emotional impact of perinatal loss upon parents but only limited research focussing specifically on the views and experiences of parents who have experienced a loss from a twin or higher order pregnancy. We undertook a qualitative study to provide an in-depth understanding of the experiences of mothers who have had a loss from a twin pregnancy and subsequently continued visiting hospital whilst their surviving twin was cared for.

**Methods:**

A qualitative study involving semi-structured interviews. Mothers were recruited from a Neonatal Intensive Care Unit and Fetal Medicine department. Fourteen interviews were carried out with mothers who had experienced a loss in pregnancy or the neonatal period and had a surviving twin on the neonatal unit. Data were analysed using a generative thematic approach.

**Results:**

The analysis identified three key themes in the accounts mothers gave of their experiences: the status of ‘special’; the importance of trust; and control and empowerment. Where the surviving co-twin remained in hospital for many weeks, mothers described the emotional support of health professionals as crucial to their wellbeing. Few mothers sought formal bereavement support, instead they kept their grief ‘*on hold’* in order to support their surviving baby. Due to the trauma of their loss, mothers reflected that they had been unable to make informed decisions, in particular in relation to the funeral of their deceased baby.

**Conclusions:**

Our study highlighted that there are a specific set of issues for mothers who have lost a baby from a twin pregnancy. Relatively small changes to practice however, made a significant difference to wellbeing during their time in hospital with a surviving twin. Findings from this research will provide insight into the needs of bereaved mothers, will inform healthcare planning and the development of care packages.

**Electronic supplementary material:**

The online version of this article (doi:10.1186/s12884-015-0579-z) contains supplementary material, which is available to authorized users.

## Background

The rates of multiple pregnancies (twins, triplets and higher order births) continue to rise as a result of increasing maternal age [1, 2] and the use of assisted reproductive techniques [3, 4]. The increased risks of adverse outcome in multiple pregnancies compared to singleton pregnancies including premature birth, congenital anomalies and perinatal deaths are well documented [5, 6]. The UK National Institute for Health and Care Excellence guidelines [7] recommend an increase in monitoring and contact with health professionals with specialist expertise for parents expecting multiples. Also acknowledged in these guidelines is the need for more psychological support for parents facing a high risk pregnancy.

There is a growing body of literature exploring the emotional impact of perinatal loss [8–13]. Henley and Schott [14] acknowledge the importance of providing sensitive emotional care that will impact upon significant life-long memories formed by parents at the time of the loss of their baby. Indeed, the UK Department of Health’s national strategy for children’s palliative care [15] put forward recommendations, not only for the care of the child, but for the emotional care of the wider family both before and after the child’s death. Redshaw et al. [16] suggest, however, that the effectiveness of the emotional and medical care given to parents following a perinatal loss can vary depending upon the approach of different health care professionals.

In contrast, there is limited research focussing specifically upon the experiences of parents who have suffered a loss from a twin or higher order pregnancy [17]. When a baby from a multiple pregnancy dies, it is largely the nurses on a Neonatal Intensive Care Unit (NICU) who support bereaved parents whilst surviving babies are cared for [18]. It is therefore important that staff have the skills and resources to effectively meet parents’ emotional needs. Many hospital support services are ill-equipped to deal with the specific needs of parents who suffer such a loss [19]. Existing studies recognise that these parents have a complex set of emotional needs that may differ from those of parents who lose a singleton [20–26].

Parkes [27] points out that, in the light of technological and medical advances, the loss of a baby is always an unexpected and very traumatic experience for parents. There are studies which focus specifically upon the grief experienced when parents lose a baby from a multiple pregnancy. Pector and Smith - Levitin [28], suggest that despite the intensity of their grief for the deceased multiple, parents will often be denied ‘permission’ from health professionals and family and friends to express this grief. Instead they will be encouraged to focus upon the ‘positives’ of a surviving baby. Cuisinier at al [29] compared levels of parental grief between a singleton loss and a loss from a multiple pregnancy and found that intensity of grief was the same. However, Wilson [30] argues that grieving will always be more complex when some babies die and others survive. Bryan [31] suggests that grief can be delayed for months or years whilst the focus is upon caring for survivors. Conversely, Pector and Smith-Levitin [28] draws attention to the fact that grieving for a lost twin or higher order multiple can cause an inability to attach to the survivor(s).

The aim of this qualitative study was to gain in-depth understanding of the experiences of mothers who have had a loss from a twin pregnancy. Mothers were given space to articulate, in their own words, their emotions at the time of their loss and their experiences of visiting hospital after their loss whilst their surviving twin received care.

## Methods

### Setting

Participants were recruited from both a NICU and Fetal Medicine Department of a tertiary hospital. The centre is recognised as specialising in twin births and there is a dedicated specialist twin’s midwife who supports parents with a twin or higher order pregnancy until the babies are born. The services of a social worker, a bereavement counsellor and a psychologist are also available at the centre, albeit on a part-time basis. However, bereaved parents are not automatically referred for specialist support due to the demands on these services.

### Sample

The sample consisted of 14 mothers with a twin pregnancy from which the loss of a twin had occurred either during pregnancy (five participants) or in the neonatal period (nine participants). Although the study initially included twins and higher order multiple births, the sample was dominated by cases involving twins. This was due to the fact that the cases involving higher order multiples overall were more limited and no cases were identified during the recruitment period. Within the sample, the loss of a twin was experienced between six months and two years prior to interview. Mothers were recruited using purposive sampling to ensure that the participant group reflected diversity of possible experiences rather than a statistically representative sample [32].

Participants were identified from hospital records by a senior neonatal physician. A letter of invitation was sent to each participant and an ‘opt in’ slip was included for them to return. They were also given a Participant Information Sheet which outlined the nature of the research and what was involved in their participation. Following receipt of a slip indicating consent to be contacted, a single researcher from the research team contacted the participant to discuss the study and arrange a time for interview. Prior to the interview commencing, the researcher gained written informed consent. Recruitment continued until theoretical saturation was reached. The researcher had substantial evidence from the data that validated conceptual categories, and also evidence that no new significant insights were emerging from the interviews [33].

The study focused specifically upon the experiences of mothers. Mothers who participated were given the option of bringing a partner or other family member to the interview. Fathers accompanied mothers in 5 of the interviews and analysis of their experiences will form the basis of a later paper. In 2 cases the babies’ grandmothers were present and in the remaining seven, mothers were interviewed alone. In 11 of the interviews the surviving twin was present.

### Interviews

Qualitative methodology is underpinned by an interpretivist epistemology. Interpretivism places emphasis on the subjective understandings of phenomena, which allows for a fuller exploration of lived experiences [34]. The data were collected via in-depth, semi-structured interviews. These were designed to elicit mothers’ own interpretations of their loss and the health care received related to the loss. The interviews were conducted in a manner that enabled free discussion, but used a topic guide as a prompt to ensure discussion covered relevant topics identified in the literature review. The guide was refined as data collection progressed, and was amended in light of new issues raised by parents. Mothers were given as much space as possible to identify and prioritize what was significant to them and articulate those issues in their own words. Mothers were encouraged to tell the ‘story of their loss’ beginning at the chronological point which made most sense to them. This allowed participants to contextualise their story in the light of previous events, for example, previous pregnancies or other children.

Interviews took place in a quiet room in a location (hospital, home or university) convenient for the participants and lasted approximately one hour. A digital recorder was used to record the interviews which were transcribed verbatim and anonymised. These anonymised transcripts formed the basis of the analysis.

### Analysis

Our research was data-driven; themes were generated from the content of the anonymised interview transcripts. The data were analysed using a generative thematic approach, an established approach in health care research. A thematic approach is acknowledged [34] as a flexible method of analysis which enables the production of an ordered collective picture of rich data. What must be recognised in thematic coding however, is the active role of the researcher. Ely et al. [35] point out that themes do not simply ‘emerge’ from the data, they are constructed and interpreted by a researcher within the framework of their contextual understanding of the data and initial research questions. The research did not adopt a formal grounded theory approach, but drew on some of the principles associated with grounded theory, such as the data-led nature of the analysis, and the constant comparison approach [33] to refining the coding of the data.

The analysis was an iterative process that began with the interviews themselves. After the first interview, the transcript was read, alongside listening to the recording, and initial codes identified. Several further interviews were conducted and transcribed. Coding was then revisited in a data meeting attended by JR, JRa and RG. This involved a collective analysis of key themes from a sample of the transcripts from the earlier interviews. The coding continued, using a process of constant comparison to refine the analysis. This qualitative, thematic approach enabled consideration of: the relationship between themes as interviews progressed; the robustness of initial coding in the light of further interviews; contradictions between and within themes; and the ways in which broader themes may encompass a range of more specific ones. Two team members (JR and RG) then read the interviews in full to provide the qualitative equivalent of inter-rater reliability [36]. Example quotations are used to illustrate these themes and participants are given an identifier P01, P02 etc.

### Ethics approval

Ethics approval for the study was received from the County Durham and Tees Valley Research Ethics Committee (reference: 11.NE.0058). The study was approved by the appropriate NHS Trust.

## Results

Three main themes, incorporating ten subthemes, were identified from the interviews:

Theme one - Status as special - encompasses three sub-themes that relate to the ways in which mothers conceptualised their experience as something different to the norm, even in the context of the NICU. The sub-themes are: feeling special; acknowledging bereavement and twin-ship; and coping with trauma and grief on hold.

Theme two - Trust - includes three further sub-themes that centre on the importance of trust in mothers’ experiences of interacting with staff. The sub-themes are: emotion work; continuity of information; and continuity of staffing and trust.

The third theme - Control and Empowerment – brings together four sub-themes that are linked to notions of agency as a mother in the NICU. These sub-themes are: location of care; impact of trauma on perceptions; impact of trauma on actions; and how mothers take control. Overall, the three key themes (status as special, trust, and control and empowerment) help to convey the experiences of the mothers in our sample, and help to identify the particular challenges they faced at the time and continue to live with.

Drawing on the themes outlined below, we have produced a list of ‘Recommendations for Best Practice’ for health professionals (Please see Additional file 1).

### Status as special

The experience of having your baby admitted to a NICU constitutes a deviation from the more common, dominant narratives on birth and new motherhood. For those who have twin births where one of the babies has died, and the other remains in NICU care, this feeling of deviation from the norm is further accentuated.

### i) Feeling special

The sense of difference reported in our participants’ accounts had often begun at an early stage in their pregnancy. Mothers often spoke of feeling ‘special’ when they discovered that they were expecting twins

*I was excited…I felt, how can I put it…I felt special. I felt special to be carrying two babies, you know, I really wanted my two babies who, I seen two babies on that scan, I wanted two babies’. (P03)*

The unexpected loss of a baby was a devastating blow which left mothers grieving for the loss of their ‘special’ status alongside the death of their baby. Most mothers were keen to stress that the joy of giving birth to a surviving baby from a twin pregnancy did not detract from their grief for the baby who died. They pointed to (well meaning) platitudes made by some health professionals who suggested that although they had suffered a bereavement, there was comfort to be had in having another baby who had survived*.* All of the mothers interviewed felt strongly that one baby cannot ‘replace’ another.

*‘What I got a lot of … the doctor at the time really quite upset me… she often said to me, ‘At least you’ve still got one’, and that was one of the worst things that anyone could possibly say. ‘You’ve still got [surviving twin] though’ and I know I’m really grateful I still have[surviving twin] but that’s like saying to someone that has a child of four and six and the six year old one dies, ‘well you’ve still got the other one, so that’s ok’. And it was really quite upsetting. I knew that she didn’t mean it in any nasty way’. (P10)*

For many of our mothers, watching the surviving sibling grow up actually exacerbated their grief as milestones were reached. Birthdays, for example, served as a stark reminder of their loss, typically coming within a short time period of the anniversary of the co-twin’s death. Mothers talked of delaying the surviving sibling’s birthday party so as not to celebrate on the day their dead twin was born.

*‘And [surviving twin’s birthday party] it’s a week after, it’s the Sunday after her birthday not at the weekend of her birthday because I couldn’t …I couldn’t em I just can’t, I just find her birthday a really difficult day’. (P04)*

### ii) Acknowledging bereavement and twin-ship

Mothers appreciated greatly those health professionals who endeavoured to keep the special twin identity of the surviving baby alive whilst in the NICU. This seemed to be particularly so where staff remembered and used the names of both deceased and surviving babies. However, when staff made errors, these instances were memorable for parents.

*‘One of the nurses that was least conscientious towards other people’s feelings kept calling him [surviving twin] by his brother’s name afterwards’. (P10)*

Whilst the medical focus tended to move quickly onto the care of the surviving baby, mothers did generally feel that staff were sensitive to the particular nature of their loss. For example, some nursing staff encouraged mothers to put a photo of their deceased twin on the cot of the surviving twin, or display a photograph of the twins together. Most mothers recognised that staff were keen to respond to their individual needs, and recognised that those needs could change over their time spent in hospital.

*‘They [health professionals] always wrote on the board… like in [NICU] they’d always write the name and then ‘twin 1’ or ‘twin 2’ underneath and they said, they asked us if we wanted that to be done or not done you know, so they were thinking about how[we felt]… I think what you didn’t want was, you know, as soon as [deceased twin] had died was everyone just treating [surviving twin] as if he was a singleton, because he wasn’t’. (P11)*

### iii) Coping: trauma and grief on hold

Mothers who had suffered the loss of a twin experienced a rollercoaster of emotions whilst in hospital with the surviving baby and beyond. They talked of contradictory feelings of simultaneous joy and grief, but also, in the period immediately following the loss, they overwhelmingly talked of feeling traumatised.

*‘I think I totally switched off looking back now, from the moment I got back to the hospital, I think I just went into autopilot…yes, this is not happening, because there were no tears or anything was there? Nothing at all for days’. (P09)*

Mothers also talked of the need to keep their emotions ‘*on hold’*, whilst caring for the surviving baby. As a result, a strong grief reaction often emerged weeks, months or even years after their babies were discharged from hospital. Interestingly, whilst participants talked of putting their feelings on hold in order to be strong for the surviving baby, they often talked of their focus on the survivor actually helping them to stay strong during the initial stages of their loss.

*‘We saw her [deceased twin] for some time after losing her and then our focus to help us get through, it was wanting to be out of hospital…and went up to [other hospital] to be with [surviving twin]. That was giving us some strength…being there for him’. (P09)*

There were bereavement support services available at the study hospital site, including a social worker, a psychologist and a bereavement counsellor. Most mothers reported that they had been offered formal support. However, they had generally felt that the offer came at a point that was too soon for them to want to have counselling, given the trauma of their loss and their desire to keep their feelings ‘*on hold*.’ Many mothers spoke of feeling the full emotional impact of their loss after several months and at this point sought counselling either from the hospital or through their GP.

*‘It was about six months…we went for bereavement counselling…I was suddenly feeling so bad…really angry’. (P05)*

Taken together, the findings encompassed in these three sub-themes suggest that the feeling of being ‘special’ or ‘different’ was commonplace at the start of their pregnancy right through to the present day. Their experiences of care in the NICU setting for the surviving baby, whilst supportive, seemed to reinforce that sense of difference, in terms of the ways in which their contradictory feelings were accommodated, and the ways in which their grieving did not seem to fit with the usual offers of support.

### Trust

Notions of trust and authenticity were central to the ways in which our participants accounted for their experiences of interacting with health professionals. This is not surprising, given that our participants could be considered vulnerable in their own right (in terms of emotional distress) and on behalf of their surviving baby, still sufficiently vulnerable to require NICU level care.

### i) Emotion work

Recognition of, and evaluation of, emotion work was an issue universally raised by all participants in their accounts of interactions with health professionals, in particular nursing staff. All mothers placed a high value on their relationships with those staff members who offered emotional support, and their accounts suggest that the nature of those relationships had a major impact on their experiences in the hospital. They often identified one particular health professional who had acknowledged their bereavement and spent time allowing them to talk about their loss, or simply made time for a friendly chat. This person became a familiar face that mothers felt they could relate to, rely on and trust. These particular health professionals (usually a member of nursing staff) figured prominently in parent’s accounts.

*‘One of the senior nurses… he just came over to me and said ‘we know what you’ve been through…if you ever want to talk about it or you want any, you know what I mean, we are here, we will not ram it down your throat, but we are here’. (P09)*

*‘ She [nurse]was really nice, we got a lot of support off her…she was more on our wave length, someone you can sort of feel comfortable talking with’. (P03)*

In general, mothers tended to see nurses and midwives as the health professionals who should be providing the majority of the emotional support they needed, as these were the staff they were in daily contact with. At the same time, however, when medical consultants met with mothers to discuss the progress of surviving siblings, a degree of emotion work was seen as very important as an acknowledgment of their traumatic circumstances.

*‘The nurses were very supportive…the doctors I don’t think so much, but only because they do ward rounds and they have to assess what is in front of them there and then and they are doing such an important thing aren’t they really, that’s kind of not relevant to them. I think the nurses are very good, they’d sit and listen to you talk until the cows came home which is brilliant’. (P01)*

*‘I had issues with one of the doctors… because he was like, ‘oh, she’s not going to make it, oh she’s, do you understand has a blood clot’, I knew he was doing his job, but I just don’t think that the way they get across bad news to parents is the best’. (P13)*

Participants often talked about nursing as a ‘vocation’ which subsequently led to the expectation that nurses would instinctively recognise their need for emotional support as parents, alongside the formal role of caring for their babies. Health professionals who were less emotionally accessible were perceived as ‘*just doing a job’* (P08), in that they carried out their caring duties in respect of the baby but did not seem to recognise the needs of mothers.

*‘It’s just they (nurses) they can be seen to be like ‘it’s just a job’ you know. Some of them on Special Care Baby Unit was just like that and just like, ‘it’s just their job’ you know. They come in and they do their job and go’ (P08)*

Interestingly, participants seemed to perceive health professionals who provided emotional support as being more competent at providing medical care for their surviving baby. Nursing staff who appeared more emotionally remote often left them feeling less confident about leaving their baby in their care. In this way, the perception of emotional responsiveness of staff had an impact on parents’ experiences when away from the hospital, as well as their experiences of being on the NICU itself.

*‘Especially on a night shift, I found that was when they [nurses] just didn’t, just didn’t seem to care. They were like…they just go in for a social and I used to come home nervous’. (P06)*

The views of mothers regarding the emotional accessibility of nursing staff in particular resonate with literature which highlights the value placed upon emotion work by patients [37, 38]. A tension exists, however, with the view that a genuine display of emotion by nursing staff is ‘unprofessional’ and potentially detrimental to the standard of care a patient receives [39, 40]. Nursing staff are generally expected to balance these two views by displaying ‘managed’ emotions which emphasises a ‘display’ of emotion rather than a deeper, more genuine feeling [41].

### ii) Continuity of information

The issue of trust went beyond notions of emotion work in how staff interacted with mothers. Another key area was the efficient transfer of both verbal and written information, and this issue was evident even prior to the birth itself. Whilst mothers felt that health professionals were generally very good at communicating information, in certain situations, some felt that they had missed the opportunity to gain vital information prior to the birth of their baby. Mothers who had a premature birth, for example, missed valuable information delivered at parenting and birthing classes. One participant received bedside training by nurses before she took her baby home.

‘*Yeah and all the things that if I’d gone to a parenting class that they would have taught me there … I had nothing and it’s like I don’t even know how to dress her and they [nurses] were brilliant, they showed me how to dress her, they showed me how to bath her, they showed me how to do nappies’. (P12)*

On the NICU itself, after the death of the baby, any perceived discontinuity in information made parents feel very anxious about their surviving baby’s care. Mothers often perceived their own role as one of actively enabling continuity of information, as they were at their baby’s side for large parts of the day and were able to gather information for themselves about their baby to pass onto staff. They also highly valued continuity of information on ward handovers, so that they didn’t need to point out to other health professionals that their baby was a twin.

*‘What I found really annoying was that I had to keep repeating my story to the nurses who were looking after [twin]. I had never met them before. Now in a handover, that is really very important that they know that [survivor] was a twin and I’m very delicate…it would have been in my notes’. (P08)*

Mothers also pointed out that badly written/inaccurate information was very upsetting, and undermined any existing sense of trust that had been built up. One participant who reported a very positive experience in terms of both her emotional care and the medical care of her baby, found her discharge letter deeply upsetting as her deceased twin wasn’t named, some information in the letter was inaccurate and the letter had a very impersonal tone.

*‘It was maybe a week or something after we were discharged we got a discharge letter about [deceased twin] who was referred to repeatedly as twin 2… Yeah I mean he had a name and the letter itself it was written by somebody who to our knowledge had never even laid eyes on [deceased twin] or us, and some of the information in it wasn’t even right about him erm you know, it like mixed up things about him. So I think that our big thing, [we were] really angry about it, that’s a very simple thing to sort out, if the baby’s got a name, that’s who he was but by that stage he wasn’t twin 2 anymore. At the time we just read the letter and thought, ‘I can’t believe they’ve managed to deal with the situation so well and then summarise it so badly’. (P11)*

### iii) Continuity of staffing and trust

Notions of trust are inferred in mothers’ accounts of their interactions with staff in relation to emotion work, and in terms of continuity of information. However, they also talked explicitly about notions of trust in their accounts. As they were likely to be in hospital with the co-twin often for many weeks following bereavement, they tended to build up trusting relationships with certain staff members that they had more contact with.

*‘I just loved her [nurse]…she took me under her wing as soon as I got in that hospital…you know and she just , I felt like I didn't want to let her go home that night, I just wanted her to stay with us…she’s just lovely and I trusted her implicitly’. (P06)*

As a result, mothers appreciated continuity in the nursing staff that cared for their baby. On a large and very busy NICU however, they often came into contact with many different health professionals.

‘*What I found quite hard, but this is totally understandable, is that obviously it was a different nurse looking after her [surviving twin] every day. When it was nurses that you got to know, you found it a bit easier because they knew what had happened but it felt like a lot of days it was a new nurse’. (P05)*

There was a dedicated specialist midwife at the research site who supported parents having a multiple pregnancy both medically and emotionally through their bereavement and the birth of healthy siblings. Her role was described by parents as vital to their emotional wellbeing whilst in hospital as, alongside specialist medical care and advice, she provided meaningful continuity and a ‘familiar face’.

*‘I think if we hadn’t had [dedicated midwife] it would have been much, much more difficult. Yeah, you know she was absolutely amazing all the way through and we did see her like I said, every two weeks… so we saw her quite a lot’. (P02)*

All mothers stressed the benefits of having their surviving baby cared for by staff that they had got to know over time, were aware of their bereavement and understood their babies’ care needs. They also perceived that nurses they saw more regularly had developed an emotional bond with their babies. Mothers valued very highly a show of emotion from health professionals at the loss of their baby or an expression of affection for surviving siblings. This perceived emotional attachment served to increase the trust and faith they developed in health professionals.

*‘Yeh and the doctors they loved her [surviving twin] because of the way she pulled through after all she had been through’. (P14)*

Overall, the findings in this theme suggest that trust and continuity are difficult to separate out from each other, in how mothers conceptualised their experiences. The continuity they described in their accounts does not seem to map well to an abstract, bureaucratic notion of consistency of information provision. Rather, the continuity they described is about consistency of understanding and empathy. These less tangible aspects of continuity seem to be key in how mothers cope with the emotional turmoil of caring for a live, but imperilled baby (or babies) having so recently lost the sibling that died.

### Control and empowerment

For any mother who has a baby in NICU, the lived experience of uncertainty and lack of control are hard to ignore. For those who have already lost one baby, the recent, direct and tangible experience of that uncertainty is important – bereavement is more than a theoretical possibility. In this context, it is understandable that the broader experience of lack of control in NICU may be experienced differently if it occurs in the context of a recent neonatal death of the surviving baby’s sibling.

### i) Location of care and lack of control

The location of care recurred in mothers’ accounts, in different ways, at various points in their journey. Mothers’ talked about their experiences in relation to different locations within the main hospital site. When admitted to hospital for observation or after the loss of a baby, mothers would, if possible be given a private room in the hospital. Whereas most mothers felt that being placed in a private room showed sensitivity to their loss, some felt that once they were in a private room away from the ward, they had been ‘abandoned’, suggesting that health professionals rarely came into the room to check on them.

*‘[Consultant] gave us a steroid injection and said he wasn’t quite sure what was going on but he kept us in [private room] for observation overnight, but by which time, by the time I was admitted, it was like midnight so there was nobody about. I think there was one midwife…I’ve never felt so lonely and so petrified in all me life, it was horrible, because you just don’t know what’s going on’. (P07)*

Those who didn’t stay in a private room after their loss unanimously expressed their distress at their baby being placed next to those who had had healthy twins.

*‘I’d just lost me baby and the other baby was in special care and I’m surrounded by babies when I didn’t have either of mine really…I just wanted to be on me own…it was just terrible’. (P08)*

*‘I think they were very caring and supportive but I think the only way you’d probably want a distinction made [from singleton] would be kind of kept away from people who have twins. It sounds awful but that can’t really happen in a hospital’. (P05)*

Mothers who had a twin transferred to another hospital were subsequently living away from home whilst their baby received specialist care. These mothers felt that they were not supported by either hospital in terms of practical help and found themselves having to organise childcare for siblings at home as well as dealing with financial matters. Similarly, they felt they were offered little emotional support at a time when they felt isolated, away from friends and family and anxious about their baby’s wellbeing.

*‘As soon as you get shifted [to another hospital], it’s like you fall into this little hole. No one can help you because they don’t know [who should be helping]so I had to find it all out for myself…they keep calling me ‘out of area’ and that’s what I was and as soon as you are out of area, you slip through the net’. (P01)*

Mothers also suggested that they had declined the opportunity for follow-up appointments in the hospital as they were held near the fetal or NICU where they lost their baby. Many suggested that such appointments should be held in a different part of the hospital to avoid unnecessary distress.

*‘That was hard mind, going back to fetal medicine…to the fetal medicine waiting room. Very hard, especially when you are sitting there and someone else gets bad news because you are sitting there thinking ‘that was me not so long ago’…maybe somewhere else in the hospital would have been better’. (P01)*

### ii) Impact of trauma upon actions

Mothers described feeling traumatised after the loss of their baby which impacted upon their actions and perceptions whilst in hospital.

Whilst bereaved mothers were visiting hospital following their loss, they had significant decisions to make in relation to their deceased twin. They felt, with the benefit of hindsight, that the trauma of their loss impeded their ability to digest information and advice, actually ask for what *they* wanted and make informed decisions which sometimes lead to significant regret.

*‘The night I had her [deceased baby], I was kind of put in a room on me own and no one checked on us all night, I was kind of left which…, I didn’t ask for her to be brought in…I was on my own for about twelve hours’. [Midwife] left her [deceased baby] on the ward so I could see her…in hindsight she should have been with me, yeah she was in a different…I don’t know whether they have like a chiller room…I wasn’t really sure. I mean I know she was somewhere on the ward and I knew I could get her if I wanted but I didn’t and now when I think about it, I don’t know why I didn’t’. (Crying)(P04)*

In particular, funeral arrangements had to be made at a time when they were anxious about surviving sick siblings on the ward. Despite guidance from the hospital based bereavement officer, some felt they were unable to make an informed decision at such a stressful time, or indeed understand the potential consequences of those decisions for the future.

*‘I just got rail, not railroaded but swept along with what they generally do, I just got railroaded into going for a cremation with nothing…and it’s just…I just think, I think that, I was given the options, you can have a burial. It was just kind of ‘this is what people do’ and you, when you’re just in a bit of a daze, I just think you go along with what people say and now I just think,…that’s my biggest regret, I’ve got nothing’. (P04)*

Mothers who experienced a loss in utero were sometimes given the option of making decisions before the birth of the deceased and healthy baby. This removed some of the stress of decision making (the decision to resuscitate for example) at what would already be a very traumatic time. Funeral arrangements could also be discussed in advance of the birth of a deceased baby which allowed parents the time to fully discuss and consider the implications of their decision. Those who took advantage of this option found it very useful. The participant below met the bereavement officer to discuss her wishes for a funeral and also the person who coordinated funeral arrangements before giving birth.

*‘[Planning in advance] was very helpful because I didn’t know at the time but as they said you know when everything kicks off do you really want to be making decisions and meeting new faces?’ Yeah so I met [bereavement officer] so eventually, when everything did happen, I knew her and the lady who organised the funeral arrangements … I signed all of the forms and everything before it all happened. So everything was sort of in place and it meant I didn’t have to worry about anything which I think is very good for the hospital to think that far ahead you know to give you that option’.(P01)*

### iii) Impact of trauma upon perceptions

Mothers often suggested that their bereavement impacted upon their perceptions of risk in relation to the surviving twin. They felt that whilst health professionals gave them clear and timely information, they recounted feeling over-anxious, or conversely, in denial about the health risks that their surviving baby was facing.

*‘I think they probably thought we were a bit stupid (laughs) because twenty-eight weeks is nothing, they deliver loads of babies at that but to us…we didn’t know if we were going to bring her home…they did try and put our minds at rest but I think that was just the way we were feeling [after their bereavement], it didn’t matter what anybody said really’. (P05)*

*‘They kept telling us it could happen with [poorly twin]…he could pass away, but I was ignoring them. I didn’t see it like that, I shut down, I ignored them. I looked for the positives, ‘he’s here… and even if he dies that is something we will deal with when we come to it. He’s lived for 24 hours, he has got a chance’. I mean I know at this point the senior nurse said obviously he’s having seizures and was having this that and the other but I chose to ignore that saying ‘well look, he is breathing”. (P03)*

### iv) How mothers take control

Lack of control was a common theme in the interviews, and the previous three sub-themes demonstrate this in relation to the key areas of where care is located, how mothers make decisions in the moment, and how risks were perceived in the context of uncertainty. In many aspects of their accounts, mothers felt that the trauma of their loss rendered them somewhat passive and helpless. However, they also felt a lack of ownership of their surviving baby in the critical care environment.

*‘Because when your baby’s in an incubator, what’s your natural thing to do? You can’t just pick your baby up and cuddle her, you can’t just do what you want to do with your baby so you do get a little bit detached and then you, and then you feel guilty because you don’t have that attachment. I don’t think it occurs to the nurses that you don’t feel like your baby is yours’. (P01)*

Whilst mothers acknowledged that they were sometimes unhappy with their baby’s care, they felt powerless to complain as ultimately they relied on the nurses to care for their baby.

*‘But then you think, we can’t complain because then you will be known as the complaining parents and he’s still got loads of treatments to do, so we never complained’. (P09)*

Mothers revealed, however, ways in which they attempted to gain more control within the hospital setting. These attempts included activities such as breastfeeding or doing basic caring tasks for their baby.

*‘I used a breast pump and sure enough there was some (milk) there and I thought, that was the one thing I could do for him, I couldn’t do anything else, I was completely helpless…I think that’s the thing that actually kept him alive’. (P07)*

Other ways in which mothers felt they were gaining control included: passing on information about their baby to nursing staff that they observed whilst sitting at their baby’s bedside; reading medical entries in the infant case notes or looking at nursing charts and observations in order to be fully aware of their babies’ health; watching nursing staff carry out procedures on their baby in order to recognise possible discontinuities in care; and swapping advice and information with other parents.

*‘We were there every single day a lot of the time, we were telling the nurses what had happened the previous day…albeit they do a very good job. It’s our [baby] we were saying ‘look, his stomas on that side, he doesn’t like laying on that side you know’. (P12)*

The findings in this theme suggest that although our participants were facing circumstances in which they often felt particularly powerless, they were able to identify and engage with opportunities to gain an increased sense of control over their baby’s care. However, they described feeling that their ability to engage with such opportunities was highly dependent on maintaining good relationships with the staff caring for their surviving baby.

## Discussion

This qualitative study identified a number of themes largely relating to bereaved mothers’ specific need for emotional support from health professionals whilst their surviving baby was being cared for. All participants stressed how highly they valued health professionals who acknowledged and showed sensitivity to their loss. Mothers’ emotional reliance on nurses and midwives in particular led to relationships of trust developing with staff who had cared for both the deceased and surviving baby. Thus mothers disliked discontinuity of the care team, preferring to see ‘familiar faces’ whom they perceived understood their own emotional needs and the specific needs of their baby (ies).

Our research highlights that mothers who experience a loss from a twin pregnancy have a specific set of needs which differ from parents who have experienced the loss of a singleton. Many of these needs can be met by relatively small changes to practice and sensitivity in the approach of health professionals. Practice which reflected sensitivity to bereaved parents included: placing a surviving twin away from healthy sets of twins/triplets; staff remembering and using the name of both deceased and surviving babies; recognition of the twin identity of the surviving baby; providing parents with the time and space to talk about their loss; whenever possible, providing continuity of the baby’s care team, special consideration of bereaved parents on the day of the baby’s funeral; and a sympathetic and sensitive approach by the neonatal physician when meeting to discuss the health of the surviving twin. The use of a small symbol, a blue butterfly that could be placed on the cot of a surviving twin to denote that the baby has a deceased twin was suggested by one mother. This avoids mothers having to repeatedly tell health professionals or other parents on the ward that their baby has a deceased twin.

Our research results resonated with the work of Swanson et al. [26] in placing emphasis upon the loss of a ‘special status’ felt by parents when a baby from a multiple pregnancy dies and also parents’ intense but mixed feelings of grief, joy, anxiety and depression. In our study, however, mothers also strongly stressed feeling traumatised in the early stages following their loss, leading to feelings of numbness and helplessness. These feelings are highlighted in the work of Rando [42] who points out that after a bereavement, feelings of vulnerability and helplessness are relatively common. Similarly, Parkes [43] describes ‘inhibited grief’ as a time after a loss when the bereaved may feel numb and unable to feel or express grief and pain.

Previous studies have tended to highlight grief over a consideration of trauma and its impact upon bereaved mothers’ behaviour and perceptions whilst in hospital, especially in the early stages after their loss. This study stresses the need for further research into the impact of trauma upon bereaved mothers’ behaviour, decision making and understandings of risk in relation to surviving siblings. Important and far-reaching decisions have to be made in hospital regarding the funeral of the deceased baby and often mothers reported feeling unable to make an informed decision or challenge decisions made by others. Decisions such as the burial/cremation of their baby, for example, led to lasting feelings of regret and distress. Pector [25] revealed that decisions around the funeral were often made in haste as parents were keen to focus upon surviving sick babies. The Perinatal Society of Australia and New Zealand clinical guidelines [44] recognise that trauma can impact upon decision making and emphasise that bereaved parents should be given time to process information effectively and make informed decisions.

Kollanti [45] highlights the ‘pressure to be ok’ for parents who have lost a baby from a multiple pregnancy so that they can be strong for survivors. Our study revealed that often parents felt it was important to keep ‘*grief on hold*’ and as a result this calls into question the usefulness of formal counselling services immediately after the loss and whilst parents are in hospital. Wilson et al. [30] point out that grieving will always be more complex if some babies die and others survive. Bryan [31] suggests that grief can be delayed for months or even years as parents feel they can’t find ‘space’ for their grieving alongside the care of a sick or premature surviving baby. Mothers in our study suggested that their need for formal counselling emerged months or years after discharge rather than at the time of their loss.

Another reason why grieving can be understood as ‘delayed’ is the traumatic nature of the loss of a baby which can leave parents feeling dazed and helpless. The unexpected nature of the loss of a baby can result in a more complex grieving process [27]. A delay in grieving can impact upon a mother’s emotional attachment to the surviving baby. Pector and Smith-Levitin [28] suggest that grief can encourage mothers to either reject or over-protect a surviving twin. Within our research, only one mother reported an initial inability to bond with her surviving baby after the loss of her twin.

Swanson et al. [26] and Pector [25] point to a large proportion of mothers feeling disenfranchised grief, in that they didn’t feel their bereavement was acknowledged by health professionals as it would have been for a singleton loss, who tended instead, to encourage them to focus upon the healthy baby. This perceived lack of permission to grieve adds to the complexity of their grief. Swanson et al. [26] and Cuisinier [29] found that a similar intensity of grief was felt by a mother who had lost a singleton or a multiple. A much smaller proportion of mothers in our study showed dissatisfaction in the way they were supported emotionally by health professionals, with many parents feeling that staff went out of their way to acknowledge their loss and maintain the twin identity of the survivor.

The timing of birthing and parenting classes was a significant issue raised by mothers who had experienced a premature birth and subsequently missed out on some or all of the classes. This led to considerable distress, as some parents went into labour lacking prior information on what to expect at the birth. Mothers suggested that classes should be held earlier in the pregnancy for those who are considered at a high risk of a premature birth so that they felt fully informed and prepared. Similarly, bedside education could be given in parenting classes after delivery and before the surviving baby is discharged.

Bryan [31] describes the ‘emotional nightmare’ of having sick multiples moved to a different hospital or multiples being split between two hospitals, arguing that hospitals must work to keep continuity of information for parents (who themselves may be split between hospitals) and provide emotional support. Mothers may also be recovering from the birth at the time they have to travel with their sick baby. Separating twins geographically and the subsequent death of one baby can mean that photographs were never taken of the multiples together. Cuff [46] suggests that paintings can be composed from individual photos that depict the deceased and surviving siblings together which may help the grieving process. Bryan [31] recommends regular updating of parents regarding the health status of all babies via verbal communication and also by sending photographs. Our study also revealed the emotional strain of having sick babies transferred to other hospitals but also the lack of not only emotional support, but practical support mothers receive if they fall ‘out of area’ for that hospital. Whilst mothers felt that information about their babies passed between hospitals was timely and effective, practical issues such as care for other siblings at home and redirecting financial benefits were left to mothers to organise at what was already an intensely stressful time. A participant suggested that parents ‘out of area’ should automatically be referred to hospital social workers for support and help on a range of issues.

Our study has several strengths. The semi-structured nature of the interviews allowed participants to articulate, in their own words, issues that were important to them. This enabled both breadth and depth of data as the research did not focus upon predefined areas of interest. A further strength was the impartial nature of the researcher who went into the research field with no preconceptions concerning how bereaved parents should be cared for. The viewpoint of the ‘naive stranger’ [47] is a valuable one in the research process as it allows for a sharper picture to emerge of the main issues.

The study also has some limitations. The findings of the study relate to the parameters of the research field set within one hospital, and may not be generalizable to other populations or settings. We have, however, drawn up a list of generic examples of best practice, based upon the findings of our research that could be applied across hospital settings (see Box below for recommendations of good practice). Mothers expressed a high degree of satisfaction regarding their care but it should be noted that the research site is regarded as a centre of excellence for multiple births. Parents referred often to the dedicated midwife who provided continuity, specialist care and the ‘familiar face’ which parents valued so highly. It is likely that at centres which do not have a dedicated multiples professional in place, mother’s satisfaction levels, especially in respect of continuity of care, may be lower. Mothers in our study also had access to a social worker, a psychologist and a bereavement counsellor, resources which may not be available at other sites. These specialist services are shared across the hospital and therefore the demand on their time is great. As a result, not all mothers are given a referral.

Our research largely focussed upon mothers ‘experiences around the time of their loss and during the time spent in hospital. However, mothers raised the issue of further uncertainty regarding the development of premature babies many years after leaving hospital. Physical or intellectual disability in the surviving twin can lead to a second loss for parents, their expectations of a healthy child [26]. Mothers revealed in our study that the full extent of their grief was often ‘delayed’ whilst in hospital and counselling was sought months after their loss. Further research into bereaved mothers’ (and fathers) experiences after discharge would be useful to explore whether their emotional care in hospital impacts upon the management of their bereavement in the years that follow.

Finally, mothers who have a loss from a twin pregnancy can experience their loss at different stages: in utero, at birth or neonatally. In our sample, most mothers had experienced a neonatal loss but further research looking more closely at the difference in experiences of parental loss at different stages of pregnancy would provide valuable insight.

## Conclusion

This study has highlighted the specific needs of mothers who have lost a baby from a twin pregnancy. More in-depth qualitative research into the specific practical and emotional issues faced by mothers as a distinct issue in the context of perinatal loss is needed. By enabling mothers to identify issues important to them in relation to their care in hospital, we can conclude that mothers themselves perceive their emotional and practical needs as differing from those experienced following the loss of a singleton. Many of their needs could be met by relatively small changes to practice, changes which acknowledge and are sensitive to parental grief and loss whilst surviving babies are being cared for. Whilst the needs of each mother will differ, more studies exploring the views of mothers will help to close the gap between health professionals’ perceptions of bereaved mothers’ needs and what those needs actually are.

## Additional file

Additional file 1:
**‘Recommendations for Best Practice’: A list of recommendations drawn from the data for health professionals, based upon the views and experiences of participants.**

## Abbreviations

NICU: Neonatal Intensive Care Unit

## References

[1] Beemsterboer S, Homburg R, Gorter NA, Schats R, Hompes P, Lambalk C (2006). The paradox of declining fertility but increasing twinning rates with advance maternal Age. Hum Reprod.

[2] Russell RB, Petrini JR, Damus K, Mattison DR, Schwartz RH (2003). The changing epidemiology of multiple births in the United States. Obstet Gynecol.

[3] Reynolds M, Schieve L, Martin J, Jeng G, Macaluso M (2003). Trends in multiple births conceived using assisted reproductive technology, united states, 1997–2000. Pediatrics.

[4] Tandberg A, Bjørge T, Nygård O, Børdahl PE, Skjaerven R (2010). Trends in incidence and mortality for triplets in Norway 1967–2006: the influence of assisted reproductive technologies. Int J Gynaecol Obstet.

[5] Keith LG, Papiernik E, Keith DM, Luke B (1995). Multiple pregnancy: epidemiology, gestation, and perinatal outcome.

[6] Glinianaia SV, Obeysekera MA, Sturgis S, Bell R (2011). Stillbirth and neonatal mortality in monochromic and dichorionic twins: a population based study. Hum Reprod.

[7] National Institute for Health and Care Excellence: Multiple pregnancy: The management of twin and triplet pregnancies in the antenatal period. [http://www.nice.org.uk/guidance/cg129] 22855972

[8] Hughes P, Riches S (2003). Psychological aspects of perinatal loss. Curr Opin Obstet Gynecol.

[9] Hutti M (2005). Social and professional support needs of families after perinatal loss. J Obstet Gynecol Neonatal Nurs.

[10] Gold KJ, Dalton VK, Shwenk TL (2007). Hospital care for parents after perinatal death. Obstet Gynecol.

[11] Einaudi MA, Le Coz P, Malzac P, Michel F, D’Ercole C, Gire C (2010). Parental experience following perinatal death: exploring the issues to make progress. Eur J Obstet Gynecol Reprod Biol.

[12] Flenady V, Wilson T (2011). Support for mothers, fathers and families after perinatal death. Cochrane review.

[13] Mills TA, Ricklesford C, Cooke A, Heazell AEP, Whitworth M, Lavender T (2014). Parent’s experiences and expectations of care in pregnancy after stillbirth or neonatal death: a metasynthesis. Int J Gynaecol Obstet.

[14] Henley A, Schott J (2008). The death of a baby before, during, or shortly after birth. Good practice from the parents’ perspectives. Semin Fetal Neonatal Med.

[15] Lewis M: Better care, better lives. Improving outcomes for children, young people and their families living with life limiting and life threatening conditions [http://www.dh.gov.uk/prod_consum_dh/groups/dh_digitalassets/@dh/@en/documents/digitalasset/dh_083108.pdf]

[16] Redshaw M, Rowe R, Henderson J (2014). Listening to parents after stillbirth or the loss of their baby after birth. National perinatal epidemiology unit.

[17] McGrath JM (2011). Supporting parents Who lose a child of a multiple birth: a critical review of research in the neonatal intensive care unit. Newborn Infant Nurs Rev.

[18] Lee KE (2012). Critical review of the literature: Parental grief after the loss of a multiple. J Neonate Nurs.

[19] De Kleine M, Cuisinier M, Kollee L, Bethlehem G, De Graauw K (1995). Guidance after twin and singleton neonatal death. Arch Dis Child.

[20] Bryan E (1986). The death of a new-born twin: how can support for parents be improved?. Acta Genet Stat Med.

[21] Bryan EM (1995). The Death of a twin. Palliat Med.

[22] Lewis E, Bryan EM (1988). Management of perinatal loss of a twin. BMJ.

[23] Wallbank S, Robertson N (2008). Midwife and nurse responses to miscarriage, stillbirth and neonatal death: A critical review of qualitative research. Evidence Based Midwifery.

[24] Attia L, Nolan A (2011). Caring for parents following the death of a twin: A student’s experience. Br J Midwifery.

[25] Pector EA (2004). Views of bereaved multiple-birth parents on life support decisions, the dying process and discussions surrounding death. J Perinatol.

[26] Swanson PB, Kane RT, Pearsall-Jones JG, Swanson CF, Croft ML (2009). How couples cope with a death of a twin or higher order multiple. Twin Res Hum Genet.

[27] Parkes CM (1985). Bereavement. Brit J of Psychiat.

[28] Pector EA, Smith-Levitin M (2002). Mourning and psychological issues in multiple birth loss. Semin Fetal Neonatal Med.

[29] Cuisinier M, DeKleine M, Kollee L, Bethlehem G, DeGraauw C (1996). Grief following the loss of a newborn twin compared to a singleton. Acta Paediatr.

[30] Wilson AL, Fenton LJ, Stevens DC, Soule DJ (1982). The death of a newborn twin: an analysis of parental bereavement. Pediatrics.

[31] Bryan EM (2002). Loss in higher multiple pregnancy and multifetal pregnancy reduction. Twin Res.

[32] Glaser B, Strauss L (1967). The Discovery of Grounded Theory: strategies for qualitative research.

[33] Pope C, Mays N (2006). Qualitative Research in Health Care.

[34] Braun V, Clarke V (2006). Using thematic analysis in psychology. Qual Res in Psych.

[35] Ely M, Vinz R, Downing M, Anzul M (1997). On writing qualitative research: living by words.

[36] Silverman D (2006). Interpreting qualitative data methods for analysing talk, text and interaction.

[37] Phillips S (1996). Labouring the emotions: expanding the remit of nursing work?. J Adv Nurs.

[38] Mitchell D, Smith P (2003). Learning from the past: emotional labour and learning disability nursing. J Learn Disabil.

[39] Mc Queen A (2004). Emotional intelligence in nursing work. J Adv Nurs.

[40] Henderson A (2001). Emotional labour and nursing: an under-appreciated aspect of caring work. Nurs Inq.

[41] Bolton SC (2001). Changing faces: nurses as emotional jugglers. Sociol Health Ill.

[42] Rando T (1992). The increasing prevalence of complicated mourning: the onslaught is just beginning. Omega-J Death Dying.

[43] Parkes CM (1965). Bereavement and mental illness. Brit J Med Psych.

[44] The Perinatal Society of Australia and New Zealand: *Clinical Practice Guidelines for Perinatal Mortality,* 2009 (Second Edition) [http://www.stillbirthalliance.org.au/doc/Section_1_Version_2.2_April_2009.pdf]

[45] Kollanti J (2002). The context and long-term impacts of multiple birth loss: a peer support network perspective. Twin Res.

[46] Cuff J (2002). Personal experiences of bereaved twins, parents of twins and their carers. Twin Res.

[47] Latour B, Woolgar S (1979). Laboratory life. The social construction of scientific facts.

