# Supplementary material for: Mothers’ perspectives on the perinatal loss of a co-twin: a qualitative study
Source: BMC Pregnancy Childbirth. 2015 Jul 2;15:143. doi: 10.1186/s12884-015-0579-z (PMC4488109; doi:10.1186/s12884-015-0579-z)
Supplement: Additional file 1: — ‘Recommendations for Best Practice’: A list of recommendations drawn from the data for health professionals, based upon the views and experiences of participants. [file 12884_2015_579_MOESM1_ESM.docx]

**Additional File 1:**

**Recommendations for Best Practice**

**Acknowledging Bereavement**

- It is important to mothers that health professionals fully acknowledge parental grief at the loss of a twin whilst simultaneously focussing upon the care of the survivor.
- Mothers value very highly health professionals who allow them time to talk about their loss and refer to the names of both their surviving and deceased twin.
- Flexibility is needed when caring for bereaved mothers as each individual copes with loss in a different way.
- Health professionals should endeavour to preserve the twin identity of a surviving twin, if the parents so wish.
- An emblem, such as a butterfly, could be placed on the surviving twin’s cot which signifies to other health professionals that the baby has a twin which has died.

**Trauma and Grief**

- Health professionals should recognise that the traumatic nature of their loss can impact upon mother’s ability to process information or make decisions in respect of the surviving baby.
- Information given to parents regarding their surviving twin should be revisited at a later time to make sure that the information has been fully understood.
- Mothers should be carefully guided through options for the funeral of their deceased twin and given time to make decisions. This could avoid lasting feelings of regret.
- The complex nature of grieving when one baby dies and another survives should be acknowledged when consideration of the relevance of professional bereavement services to mothers.
- Mothers often declined to return to the hospital for follow-up meetings as they did not wish to return to the place where they had lost their baby. Appointments after discharge should be held away from the neonatal ward.
- The offer of counselling should be kept open to mothers after discharge as a grief response can be delayed by months or even years.

**Information**

- Any changes to the surviving baby’s care plan should be fully explained to parents before those changes take place.
- Wherever possible, continuity of the care team is important for bereaved mothers. This provides ‘familiar faces’ for mothers with whom they build up relationships of trust during their time in hospital.
- Mothers expecting twins are at a bigger risk of premature birth. This means that birthing and parenting classes can be missed. Bedside training could be sought for mothers if possible before birth occurs.
- Decisions regarding the birth of a deceased twin can be discussed with a mother before the birth actually happens. These decisions can be recorded clearly in the mother’s notes.
- Health professionals should ensure that the loss of a twin is recorded in hospital notes and similarly, that notes are fully read.

**Hospital locations**

- Mothers who have twins placed in different hospitals should be supported emotionally and practically by a social worker or other allied health professional. Continuity of communication is vital between the hospitals for keeping mother up to date with the care of both her sick babies.
- Placing a bereaved mother and her surviving twin next to parents with healthy twins should be avoided if possible.
- Bereaved mothers who are given a side room on a ward should be checked upon regularly by medical staff to avoid mothers feeling ‘abandoned’.
- A dedicated healthcare professional for twin and multiple births is recommended to provide continuity of care for mothers from conception through to neonatal care and beyond discharge. This role provides mothers with access to specialised information from practical to medical and importantly a sense of continuity, trust and emotional support.
